# *Belonolaimus longicaudatus* management using metam potassium and fluensulfone in potato

**DOI:** 10.2478/jofnem-2023-0028

**Published:** 2023-07-06

**Authors:** Zane J. Grabau, Chang Liu, Pablo A. Navia Gine

**Affiliations:** Entomology and Nematology Department, University of Florida, 1881 Natural Area Drive, Gainesville, FL 32611, United States; 1728 Shoreside Circle, Wellington, FL 33414, United States

**Keywords:** 1,3-Dichloropropene, *Belonolaimus longicaudatus*, fluensulfone, fumigant, metam potassium, nematicide, nematode community, potato, *Solanum tuberosum*, sting nematode

## Abstract

*Belonolaimus longicaudatus* (sting nematode) is an important pest in Florida potato production and is managed primarily by fumigation using 1,3-dichloropropene (1,3-D). Other effective nematicides are needed for more flexibility in managing this pest. The objective of this study was to evaluate fluensulfone, metam potassium, and mixtures of the two products, relative to 1,3-D and untreated control, for efficacy at managing sting nematode, and for non-target effects on free-living nematodes in potato. To test this objective, a small-plot field experiment was conducted in northeast Florida in 2020 and repeated in 2021. Metam potassium fumigation (390 kg a.i./treated ha)—with or without fluensulfone—managed sting nematode soil abundances but was phytotoxic to potato. Strategies that mitigate metam potassium phytotoxicity, such as reduced application rates, are needed before efficacy of metam potassium in this system can be determined. As a preplant soil spray, fluensulfone alone (403 g a.i./treated ha) did not manage sting nematode abundances and had an inconsistent effect on yield. Fumigation with 1,3-D (88.3 kg a.i./treated ha) was the only treatment that consistently managed sting nematode and increased potato yield. Nematicides did not consistently affect free-living nematodes.

Potato (*Solanum tuberosum*) production is an important industry in the United States, with the 2021 potato crop worth 4.17 billion dollars (USDA-NASS, 2022). Potato production is also a valued industry in Florida, with 8,500 hectares of production, and a total crop value of 95 million dollars in 2021 (USDA-NASS, 2022). While this is a relatively small proportion of U.S. production (377,000 hectares), it is a substantial portion of spring potato production, and Florida is the leading potato-producing state in the Southeast (USDA-NASS, 2022). Florida potato production is exclusively short season (approximately 100 days) types grown for the fresh market and chipping industry (USDA-NASS, 2022).

Sting nematode (*Belonolaimus longicaudatus*) is widespread in Florida potato production—particularly in the important production area of northeast Florida—and highly damaging (Crow et al., 2000b). Crop rotation consisting of continuous winter potato followed by summer sorghum–sudan grass (*Sorghum* x *drummondii*) cover crop is standard in northeast Florida potato production, and sting nematode increases on both of these crops (Crow et al., 2000a, 2001). This rotation, along with ubiquitous sandy soils, helps make the sting nematode prevalent in Florida potato production.

Management of sting nematode—and other plant-parasitic nematodes—on potato in Florida relies heavily on nematicide application. There are a limited number of nematicides available, so additional effective nematicides are always needed to reduce dependency on current options. For sting nematode management in potato, fumigation with 1,3-dichloropropene (1,3-D) is the most common tactic, and growers may also apply chloropicrin in the fumigant mixture because it improves soil-borne disease management (Csinos et al., 2000; Gilreath et al., 2004). More recently, the non-fumigant nematicide fluensulfone was introduced and has shown efficacy at managing sting nematode in Florida potato (Grabau et al., 2019), although fluensulfone is not widely adopted in commercial production. Another nematode management option is fumigation with metam salts (metam potassium or metam sodium), which have been available for many years and are used by some Florida potato growers. Metam is a broad-spectrum fumigant with activity against fungal pathogens and weeds as well as nematodes (Csinos et al., 2000; Gilreath et al., 2004; Desaeger et al., 2017). However, despite being used commercially, there is need for formal evaluation of metam efficacy for managing sting nematodes in potato, due to a lack of published reports.

The only reports on efficacy of metam salts against sting nematode are on strawberry (*Fragaria* x *ananassa*) and tomato (*Solanum lycopersicum*) in Florida (Santos et al., 2006; Gilreath et al., 2008; Watson and Desaeger, 2019). Results have been mixed in those systems with metam salts variously being effective (Santos et al., 2006), ineffective (Watson and Desaeger, 2019), or effective only in combination with chloropicrin (Gilreath et al., 2008). Metam products have been evaluated more broadly against root-knot nematodes in the Southeast. Metam can be effective for root-knot nematode management (Csinos et al., 2000; Desaeger and Csinos, 2006), but is considered less consistent for nematode management than 1,3-D in the sandy soils common in the Southeast (Desaeger et al., 2017; Watson and Desaeger, 2019).

A potential tactic for improving nematode management by metam potassium (MP) is combining MP with an effective nematicide such as fluensulfone (FL). This may also increase grower adoption relative to either product alone, as a successful mixture would have efficacy against a wider spectrum of pests. Fumigants are commonly combined or applied in tandem for this purpose (Csinos et al., 2002; Desaeger et al., 2017). Applying a non-fumigant nematicide at or after planting to supplement preplant fumigation has also been tested, albeit with varying levels of improved efficacy relative to fumigation alone (Desaeger et al., 2017; Watson and Desaeger, 2019; Grabau et al., 2021). Mixing a fumigant with a non-fumigant nematicide is more novel, but mixtures of fluensulfone and MP effectively managed sting nematode and southern root-knot nematodes in initial tests in Southeast vegetable production (Navia Gine and Hajihassani, 2021).

As emphasis on sustainability and the entire soil community—including beneficial organisms—has increased, so too has the importance of considering non-target effects of pesticides, including nematicides. In particular, nematicide application can have unintended negative impacts on free-living nematodes (Waldo et al., 2019; Grabau et al., 2020). Free-living nematodes feed on fungi, bacteria, or other nematodes and can provide benefits to the soil ecosystem such as nutrient cycling (Holajjer et al., 2016; Trap et al., 2016) and pest regulation (Khan and Kim, 2005). Therefore, evaluating free-living nematodes provides important additional information about how nematicides affect the broader agroecosystem.

Based on these needs, the objectives of this study were to evaluate the efficacy of MP and a mixture of MP and FL on (1) management of plant-parasitic nematodes (2) yield response, and (3) non-target impacts on free-living nematodes in Florida potato production.

## Materials and Methods

### Site and Experimental Design

To investigate these objectives, a field experiment was conducted at the University of Florida Hastings Agricultural Education Center near Hastings, Florida (29.692, −81.441). The soil type was Ellzey fine sand (sandy, silicaceous, hyperthermic Arenic Ochraqualf) with 95% sand, 2% silt, 3% clay; and <1% organic matter. Production was on bareground, raised beds irrigated by a subsurface tile system, which is common in the region. Cropping history was continuous spring potato followed by summer sorghum–sudan grass cover crop. The experiment was conducted twice, with the first trial occurring in early 2020 and the second trial occurring in early 2021, with some establishment activities occurring in the preceding year for each trial. Experimental units were 4-row beds 19.8 m long. Center-to-center bed spacing was 101 cm. The experiment was a randomized complete block design with six replicates and a single factor: nematicide treatment. There were five treatments, including 1) untreated control (UTC), 2) MP alone (MP-A), 3) MP mixed with fluensulfone (MP+FL), 4) fluensulfone alone (FL-A), and 5) 1,3-D. Treatment rates and products are described in Table 1.

**Table 1.** Chemistries and rates for nematicide treatments.

**Treatment abbreviation**	**Product**	**Active ingredient**	**Liters product/bedded ha^a^**	**kg a.i./bedded ha**	**Method**
1. UTC	Untreated control	None	-	-	-
2. MP-A	Metam KLR^b^	Metam potassium	562	390	Fumigation
3. MP+FL	Metam KLR	Metam potassium	562	390	Fumigation (tank mix)
	NIMITZ^c^	Fluensulfone	7.0	3.36	
4. FL-A	NIMITZ	Fluensulfone	7.0	3.36	Ground spray
5. 1,3-D	Telone II^d^	1,3-dichloropropene	75	88.3	Fumigation

a Bedded acreage was 75% of broadcast acreage. In this trial, the bedded acreage was considered the acreage to which treatments were applied. Further details of treatment applications are described in the materials and methods.

b Metam KLR (Taminco, Allentown, PA)

c NIMITZ (ADAMA, Research Triangle Park, NC)

d Telone II (Dow Agrosciences LLC, Zionsville, IN)

### Fumigation application methods

Metam potassium (alone or as a mixture) and 1,3-D were applied by preplant fumigation using shank injection, but fumigation rig setup varied slightly for each product according to standard practices in the region. For 1,3-D, the fumigation rig had 1 shank per bed (101 cm spacing between shanks). The MP-A and MP+FL treatments were applied using 3 shanks per bed that were spaced 13 cm apart. More shanks per bed were used for MP-A and MP+FL to ensure adequate bed coverage due to relatively low volatility of MP (Gertzl et al., 1977). For MP+FL, FL and MP were combined in the containing tank of the fumigation rig in the correct proportions before application. Fumigation was conducted 25 or 26 days before planting each season (Table 2). In all cases, nematicides were applied to flattened beds that had been tilled with a rotary chopper and subsoiler after summer sorghum–sudan grass production. Fumigants were delivered approximately 15 cm deep in flattened beds and immediately followed by a second tractor equipped with angled disc implements that formed a hilled bed of loose soil in fumigant-treated plots. The fumigation rig that delivered 1,3-D was also equipped with angled discs directly on the rig. After bed formation, final fumigant placement was at least 30 cm below the tops of beds. Bed formation is the standard practice for containing fumigants in the soil—as required on fumigant labels—in potato production in the area.

**Table 2.** Schedule for data collection and trial establishment. Numbers in parentheses are days before (negative) or after (positive) planting.

**Event**	**Year 1 (2019–2020)**	**Year 2 (2020–2021)**
Preplant soil sampling	18 Dec (−38)	14 Dec (−30)
Nematicide application	2 Jan (−25)	18 Dec (−26)
Beds tilled and re-formed	13 Jan (−14)	5 Jan (−8)
Planting	27 Jan (0)	13 Jan (0)
Stand count 1	26 Feb (30)	18 Feb (36)
Stand count 2	4 Mar (37)	26 Feb (44)
Vigor rating 1	26 Feb (30)	18 Feb (36)
Vigor rating 2	4 Mar (37)	26 Feb (44)
Vigor rating 3	18 Mar (52)	3 Mar (50)
Vigor rating 4	–	12 Mar (59)
Midseason soil sampling	4 Mar (37)	26 Feb (44)
Harvest soil sampling	8 May (103)	14 Apr (92)
Harvest	11 May (106)	21 Apr (99)

### Fluensulfone application

For the standalone treatment, FL was sprayed on top of the soil as a broadcast application using a commercial tractor and boom sprayer. Immediately following application, fluensulfone was incorporated to 15 cm by tilling with a rotary chopper. Fluensulfone was applied on the same day as fumigant treatments, either 25 or 26 days before planting (Table 2). On the same day, following application of all treatments, individual fluensulfone and untreated control plots were angle-disced to form beds. This ensured that all plots were bedded the same number of times. Then, the entire trial was angle-disced to form uniform beds.

### Crop production

One to two weeks before planting (Table 2), preplant fertilizer was applied, followed by incorporation with a rotary chopper after which beds were re-formed. In each trial, “Red LaSota”, a red fresh market potato common in the area, was grown. Seed potatoes were planted in January (Table 2) using a mechanical planter. The planter was equipped with a tillage implement that opened a furrow in the beds. Seed pieces were planted at approximately 21 cm spacing in the seed furrow. After the trial was planted, a tank-mix of standard fungicide (azoxystrobin at 191 g a.i./ha) and insecticide (fipronil at 98 g a.i./ha) treatments were sprayed in furrow. The furrow was then closed, and the beds were formed again. Aside from nematicide treatments, each year, the whole trial was managed uniformly with full herbicide, fungicide, and insecticide regimes as standard for the area.

### Environmental conditions following nematicide application

Environmental conditions can affect nematicide efficacy, and soil moisture and temperature requirements are listed on fumigant labels. Soil moisture was not quantified formally at the site but was acceptable at fumigation for both the 2020 and 2021 trials based on the USDA-established feel test (USDA-NRCS, 2005). Early season soil temperature, air temperature, and rainfall are described in Table 3 based on information from the Florida Automated Weather Network (https://fawn.ifas.ufl.edu/) station at the study site. Briefly, the 2020 trial was very dry between fumigation and planting, whereas the 2021 trial received more regular rainfall during that period (Table 3). In both years soil temperatures were acceptable for fumigation, with higher temperatures in 2020 than 2021.

**Table 3.** Environmental conditions during the weeks before and after nematicide application for trials in 2019–2020 and 2020–2021

**Time Period^a^**	**2019–2020**			**2020–2021**		
	
		**Temperature (°C)**			**Temperature (°C)**	
			
	**Rainfall^b^**	**Soil^c^**	**Air**	**Rainfall**	**Soil**	**Air**
2 WBA	7.54	16.9	16.0	0.53	16.3	12.0
1 WBA	1.02	18.6	18.3	0.69	16.7	15.2
Day of application	0	16.8	16.5	0	14.9	7.3
1 WAA	0.53	16.4	14.0	2.54	15.5	13.3
2 WAA	0.00	18.4	21.1	0.25	14.3	13.9
3 WAA	0.00	17.0	13.1	0.23	15.9	14.2
4 WAA^d^	0.03	16.1	13.2	0.20	14.2	11.2
5 WAA	1.42	16.3	15.4	0.13	13.3	11.3
6 WAA	0.08	17.3	17.5	2.51	16.1	15.8
7 WAA	0.86	18.5	18.1	0.51	13.4	11.1
8 WAA	1.93	17.1	14.0	6.73	16.7	18.1
9 WAA	0.20	16.9	16.4	5.66	18.2	17.7
10 WAA	0.08	17.3	17.5	2.51	16.1	15.8

a WBA and WAA are weeks before nematicide application and weeks after nematicide application, respectively.

b Rainfall (cm) is total for the respective week or day of nematicide application.

c Temperatures are mean for the week or day.

d Potatoes were planted during 4 weeks after application, at 25 or 26 days after application in 2020 and 2021, respectively.

### Potato harvest and yield collection

Potatoes were grown for approximately 100 days before harvest in late April or early May (Table 2). From the central 2 rows of each plot, potatoes were harvested from 6.1 m of each row using a single-row mechanical harvester. Separately by plot, harvested potatoes were washed and graded using a mechanical size sorter. In 2021, the mechanical size sorter was supplemented by human intervention to remove culls with external quality defects. In 2020, quality culls were not removed or measured due to workforce restrictions during the COVID-19 pandemic. For each plot, potatoes were weighed for each grade category. Size classes were A3 (8.3–10.2 cm diameter), A2 (6.4–8.29 cm diameter), A1 (4.8–6.39 cm diameter), B (3.8–4.69 cm diameter), and C (1.3–3.79 cm diameter) based on local and USDA guidelines (USDA-AMS, 2011). No potatoes with diameter greater than 10.2 cm (class A4) were observed in either trial. All class A potatoes were considered marketable (USDA-AMS, 2011). In 2021, the categories of quality-related culls included tubers that were rotted, green (sun damage), had growth cracks, or were misshapen. In addition to weight by each cull category, the total weight of quality culls and the grand total cull weight (quality culls plus undersize tubers) was reported in 2021. For both years, total cull weight of tubers that were under marketable size (class B and C), as well as grand total yield is reported. For each plot, 20 tubers were quartered and inspected for internal defects in each trial, but there were negligible defects, so this data was not reported.

### Plant stand and vigor assessment

Plant stand was estimated by counting emerged shoots from 2.4 m in each of the central 2 rows of each plot and converted to stand per 3 m row for reporting. In both 2020 and 2021, plant stand was estimated twice: (1) when shoots started to emerge and (2) when all shoots had fully emerged (Table 2). Plant vigor was rated visually 3 to 4 times during the middle of the growing season (Table 2). Vigor was assessed from central 2 rows of each plot based on a 0 to 100 scale where 0 indicates all plants dead, 25 is a very poor crop, 50 is a below-average crop, 75 is an average crop, and 100 is an exceptionally excellent crop for the given time point. Vigor assessment was based primarily on the canopy height and size. Additionally, symptoms of phytotoxicity were observed in the 2020 trial and were rated at 37 days after planting (DAP). These symptoms are described further in the results.

### Nematode quantification

Nematode soil abundances in each plot were quantified before nematicide application (preplant), at midseason (6 weeks after planting), and near harvest each year (Table 2). Using an Oakfield tube, 12 soil cores to 25 cm depth were collected from the central 2 beds of each plot, with samples collected near plant roots at midseason and harvest. Soil was homogenized manually, and nematodes were extracted using the sucrose-centrifugation method (Jenkins, 1964). Plant-parasitic nematodes were identified morphologically and quantified by microscope. Total free-living nematode abundances were also quantified, but taxonomic identification of free-living nematodes was not completed.

### Statistical analysis

Each variable was analyzed separately for each trial and sampling date using one-way ANOVA. Before completing ANOVA, response variables were transformed, if needed, to meet assumptions of homogeneity of variance using Levene’s test (Levene, 1960) and normality of residuals based on graphing (Cook and Weisburg, 1999). Nematicide effects were considered significant at α=0.05 in ANOVA. Nematicide treatment means were separated by Fisher’s protected LSD (α=0.05) if main effects were significant in ANOVA. Analyses were conducted in R statistical software (version 3.4.4, The R Foundation for Statistical Computing, Vienna, Austria).

## Results

### Plant stand and vigor

**Figure 1:**
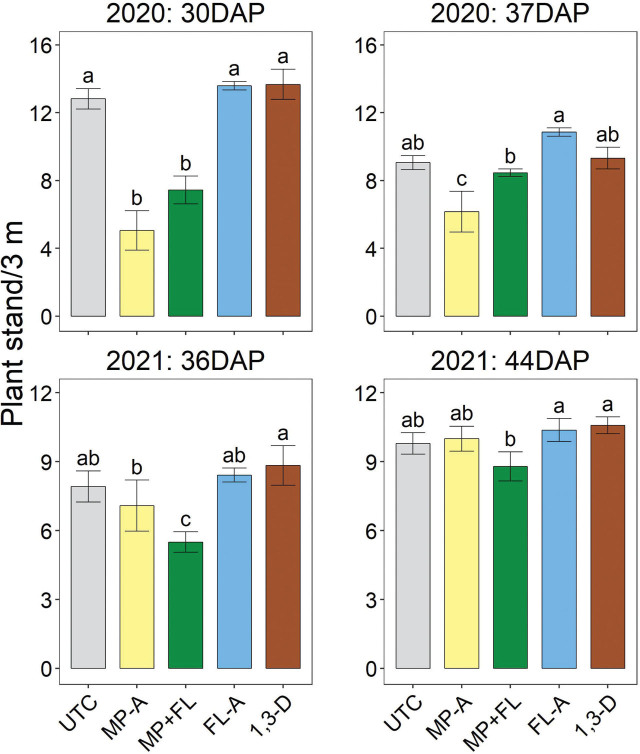
Potato plant stand as affected by nematicide application in 2020 and 2021. Timing indicated in days after planting (DAP). Bars and brackets represent means (N=6) and standard errors, respectively. “UTC”, “MP-A”, “MP+FL”, “FL-A”, and “1,3-D” represent untreated control, metam potassium alone, metam potassium mixed with fluensulfone, fluensulfone alone, and 1,3-dichloropropene nematicide treatments, respectively. Within each subfigure, means with different letters are significantly different (Fisher’s protected LSD, α=0.05).

In both 2020 and 2021, MP-A or MP+FL generally significantly reduced plant stand, particularly for the earlier assessment conducted at seedling emergence (Fig. 1). In 2020, MP-A or MP+FL significantly decreased plant stand at 30 DAP relative to all other treatments. In 2020, stand at 37 DAP—when plants had fully emerged—was significantly less for MP-A than any other treatment and less for MP+FL than FL-A. At 36 DAP in 2021, MP+FL significantly reduced stand relative to all other treatments and MP-A reduced stand relative to 1,3-D. By 44 DAP, MP+FL had less stand than 1,3-D or FL-A, but there were no other significant differences among treatments.

### Plant vigor and phytotoxicity

**Figure 2:**
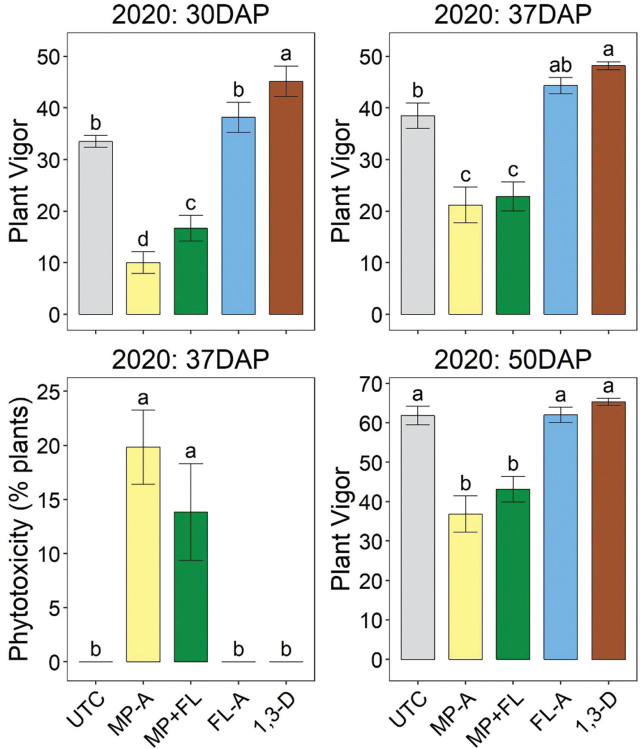
Potato plant vigor and phytotoxicity during production 2020 as affected by nematicide application. Timing indicated in days after planting (DAP). Plant vigor is a visual rating on a 0–100 scale with 100 best. Bars and brackets represent means (N=6) and standard errors, respectively. “UTC”, “MP-A”, “MP+FL”, “FL-A”, and “1,3-D” represent untreated control, metam potassium alone, metam potassium mixed with fluensulfone, fluensulfone alone, and 1,3-dichloropropene nematicide treatments, respectively. Within each subfigure, means with different letters are significantly different (Fisher’s protected LSD, α=0.05).

**Figure 3:**
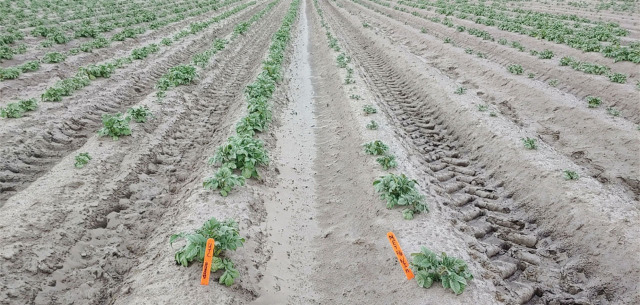
Reduced stand and plant vigor in metam+fluensulfone treatment (right) compared with untreated control (left) at 36 days after planting in 2020.

**Figure 4:**
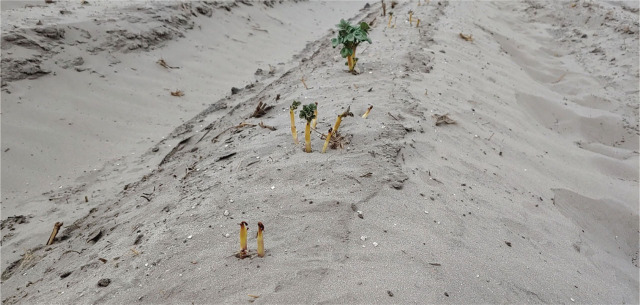
Symptoms of phytotoxicity on emerging potato shoots in treatments with metam potassium alone or in combination with fluensulfone at 36 days after planting in 2020. There is browning at tips of shoots and leaf unfolding is delayed.

In 2020, MP-A and MP+FL significantly reduced plant vigor compared with any other treatment at each assessment date (Figs. 2, 3). Qualitative, visual symptoms of phytotoxicity—particularly browning of shoot tips and delayed leaf emergence producing a burnt appearance—were observed for those treatments in 2020 beginning at 30 DAP (Fig. 4). When phytotoxicity was rated at 37 DAP in 2020, symptoms were only observed for MP-A and MP+FL (Fig. 2). By the next assessment date (50 DAP), no browning was observed on leaves.

**Figure 5:**
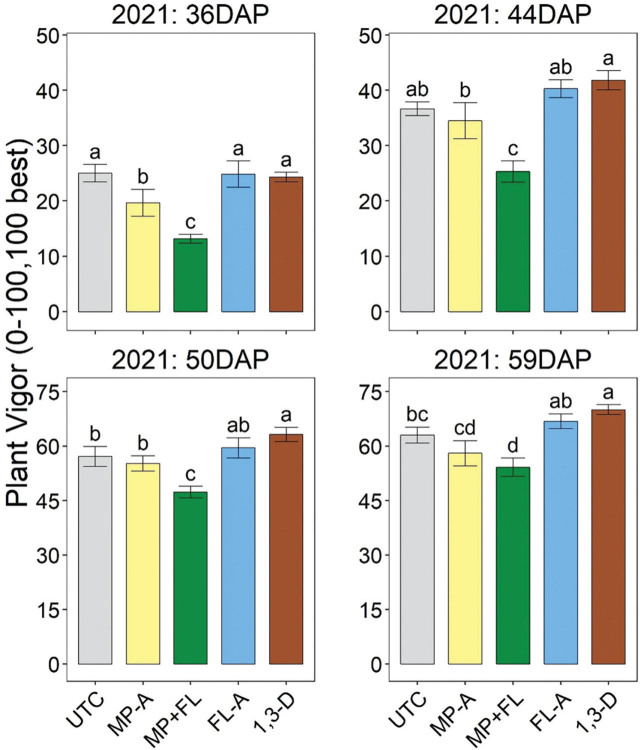
Potato plant vigor during production in 2021 as affected by nematicide application. Timing indicated in days after planting (DAP). Plant vigor is a visual rating on a 0–100 scale with 100 best. Bars and brackets represent means (N=6) and standard errors, respectively. “UTC”, “MP-A”, “MP+FL”, “FL-A”, and “1,3-D” represent untreated control, metam potassium alone, metam potassium mixed with fluensulfone, fluensulfone alone, and 1,3-dichloropropene nematicide treatments, respectively. Within each subfigure, means with different letters are significantly different (Fisher’s protected LSD, α=0.05).

In 2021, for all assessment dates, plant vigor was significantly less for MP+FL than any other treatment, except that it was similar to MP-A at 59 DAP (Fig. 5). Plant vigor was also significantly less for MP-A than 1,3-D at each assessment date and less than FL-A at 36 and 59 DAP. Later in the season (50 and 59 DAP), plant vigor was significantly less for UTC than 1,3-D.

### Potato yield

**Figure 6:**
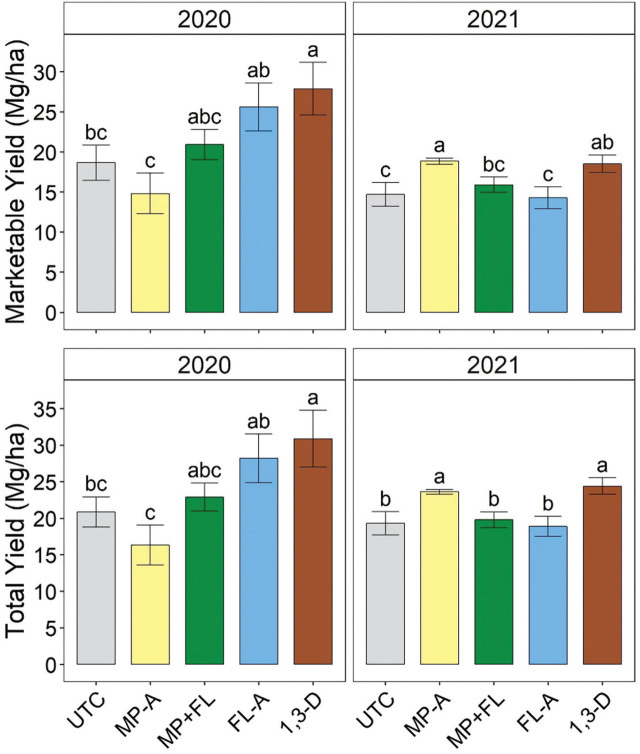
Marketable and total yield as affected by nematicide application in 2020 and 2021. Marketable yield is USDA size A tubers without defects. Total yield includes marketable yield and culls due to size or quality. Bars and brackets represent means (N=6) and standard errors, respectively. “UTC”, “MP-A”, “MP+FL”, “FL-A”, and “1,3-D” represent untreated control, metam potassium alone, metam potassium mixed with fluensulfone, fluensulfone alone, and 1,3-dichloropropene nematicide treatments, respectively. Within each subfigure, means with different letters are significantly different (Fisher’s protected LSD, α=0.05).

In 2020, total and marketable yields were significantly greater for 1,3-D than MP-A and UTC (Fig. 6). Total and marketable yields were also significantly greater for FL-A than MP-A in 2020. Class A1 and A2 yields were significantly affected by nematicides in 2020 and followed the same trends as marketable yield (Table 4). No other size category was significantly affected by nematicide treatments in 2020.

**Table 4.** Potato tuber yield (kg/ha) by grade and nematicide treatment.^a^

**Grade**	**C^b^**	**B**	**A1**	**A2**	**A3**	**Size culls^c^**
**Nematicide Treatment**	**2020**					
Untreated control	805	1,408	4,965 bc	13,243 bc	451	2,213
Metam potassium	637	849	3,904 c	10,613 c	303	1,486
Metam+Fluensulfone	839	1,147	6,014 abc	14,429 abc	468	1,986
Fluensulfone	773	1,832	7,954 ab	17,112 ab	547	2,606
1,3-dichlorpropene	1,039	1,969	8,948 a	18,884 a	54	3,008
**Nematicide Treatment**	**2021**					
Untreated control	1,394 ab	1,914 b	6,934	7,782 c	0	3,308 b
Metam potassium	1,267 b	1,940 b	7,266	11,596 a	0	3,208 b
Metam+Fluensulfone	1,145 b	1,763 b	6,845	9,066 bc	0	2,908 b
Fluensulfone	1,272 b	1,998 b	6,729	7,577 c	0	3,270 b
1,3-dichlorpropene	1,585 a	2,511 a	7,645	10,886 ab	0	4,095 a

a Different letters in the same column indicate significantly different means based on Fisher’s protected LSD at α = 0.05.

b Tuber grades of C, B, A1, A2, and A3 include harvest potatoes with diameters of 1.3–3.7, 3.8–4.7, 4.8–6.3, 6.4–8.3, and 8.4–10.2 cm respectively. Grades A1, A2, and A3 are considered marketable.

c Size culls include grade C and B tubers

In 2021, total yield was significantly greater for MP-A and 1,3-D than any other treatment. Marketable yield in 2021 followed a similar trend except that MP+FL had similar yield to 1,3-D. In 2021, there were no size A3 potatoes and A1 yield was not significantly affected by nematicide treatments (Table 4). Class A2 yield was significantly affected by nematicides in 2020, with yield significantly greater for MP-A than each treatment except 1,3-D and significantly greater for 1,3-D than UTC or FL-A. In 2021, the only year quality-related culls were reported, rotted and grand total cull weights were significantly affected by nematicides and generally followed the same trends as marketable yield (Table 5). In contrast, green cull weight was significantly greater for MP-A than UTC, FL, or 1,3-D and greater for MP+FL than UTC or FL-A. Growth crack, misshapen, or total quality culls were not significantly affected by nematicide treatments in 2021.

**Table 5.** Weight (kg/ha) of tubers culled due to quality defects by category in 2021 season.^a^

**Nematicide Treatment**	**Rotted**	**Green**	**Growth crack**	**Misshapen**	**Total quality culls^b^**	**Grand total culls^c^**
Untreated control	469 ab	72 c	443	305	1,289	4,598 b
Metam potassium	237 bc	457 a	575	291	1,561	4,768 b
Metam+Fluensulfone	291 abc	299 ab	335	41	967	3,874 c
Fluensulfone	145 c	65 c	941	168	1,319	4,590 b
1,3-dichlorpropene	545 a	74 bc	1,127	37	1,782	5,877 a

a Different letters in the same column indicate significantly different means based on Fisher’s protected LSD at α = 0.05. Due to COVID-19 restrictions, tuber defect culls were not measured in 2020

b Total quality culls is the sum of tubers in the rotted, green, growth crack, and misshapen categories.

c Grand total culls is the sum of size and quality culls.

### Plant-parasitic nematodes

**Figure 7:**
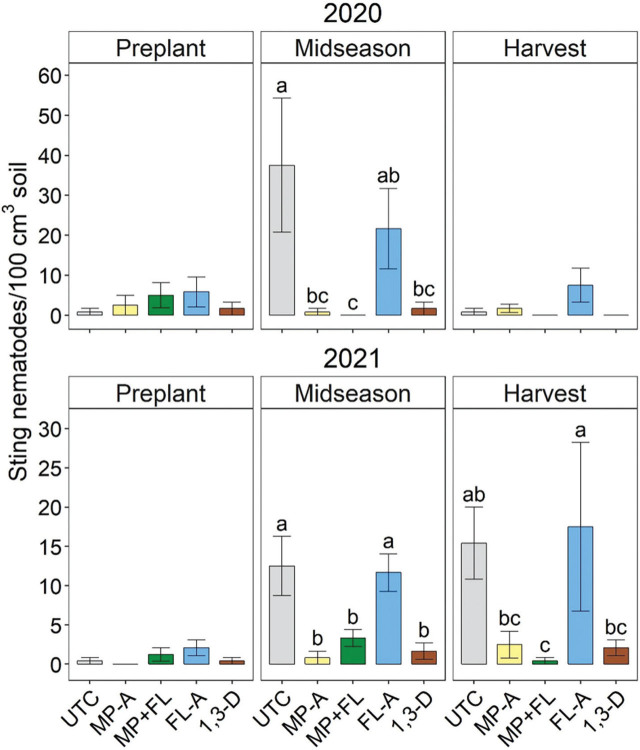
Sting nematode soil population densities at preplant (before fumigation), midseason (37 to 44 days after planting), and harvest in 2020 and 2021 as affected by nematicide treatments. Bars and brackets represent means (N=6) and standard errors, respectively. “UTC”, “MP-A”, “MP+FL”, “FL-A”, and “1,3-D” represent untreated control, metam potassium alone, metam potassium mixed with fluensulfone, fluensulfone alone, and 1,3-dichloropropene nematicide treatments, respectively. Within each subfigure, means with different letters are significantly different (Fisher’s protected LSD, α=0.05). effects for a given season (ANOVA, *P* < 0.05).

Each year, sting nematode abundances were relatively low before fumigation and did not vary significantly based on upcoming treatments (Fig. 7). Sting nematode abundances were generally greater for UTC or FL-A than treatments including MP or 1,3-D at midseason in 2020 and 2021 as well as harvest in 2021, but exact significance of treatment separation varied by season (Fig. 7). Sting nematode abundances were low and unaffected by nematicides at harvest in 2020.

**Figure 8:**
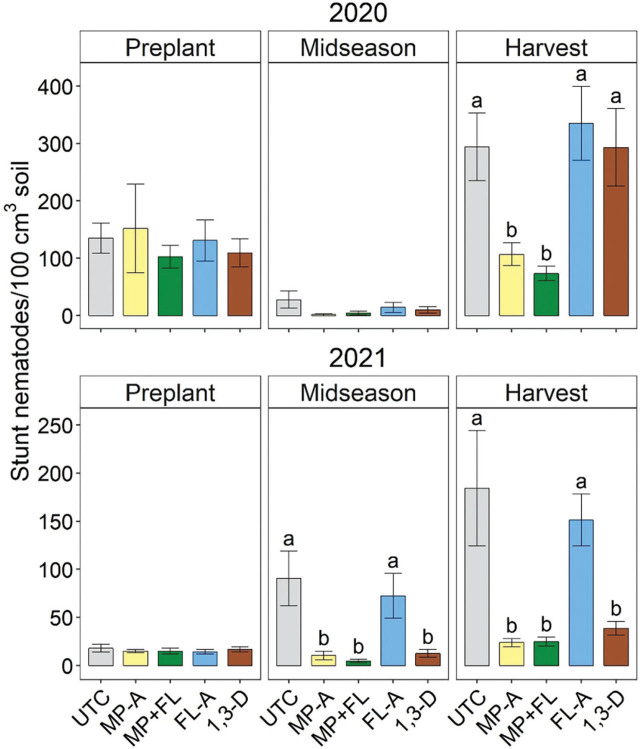
Stunt nematode soil population densities at preplant (before fumigation), midseason (37 to 44 days after planting), and harvest in 2020 and 2021 as affected by nematicide treatments. Bars and brackets represent means (N=6) and standard errors, respectively. “UTC”, “MP-A”, “MP+FL”, “FL-A”, and “1,3-D” represent untreated control, metam potassium alone, metam potassium mixed with fluensulfone, fluensulfone alone, and 1,3-dichloropropene nematicide treatments, respectively. Within each subfigure, means with different letters are significantly different (Fisher’s protected LSD, α=0.05).

At harvest in 2020, stunt nematode abundances were significantly greater for UTC, FL-A, or 1,3-D than MP-A or MP+FL (Fig. 8). At midseason and harvest in 2021, stunt nematode abundances were significantly greater for UTC or FL-A than any other nematicide treatment. Stunt nematode abundances were not significantly affected by nematicides at midseason in 2020 or preplant in either year.

### Free-living nematodes

**Figure 9:**
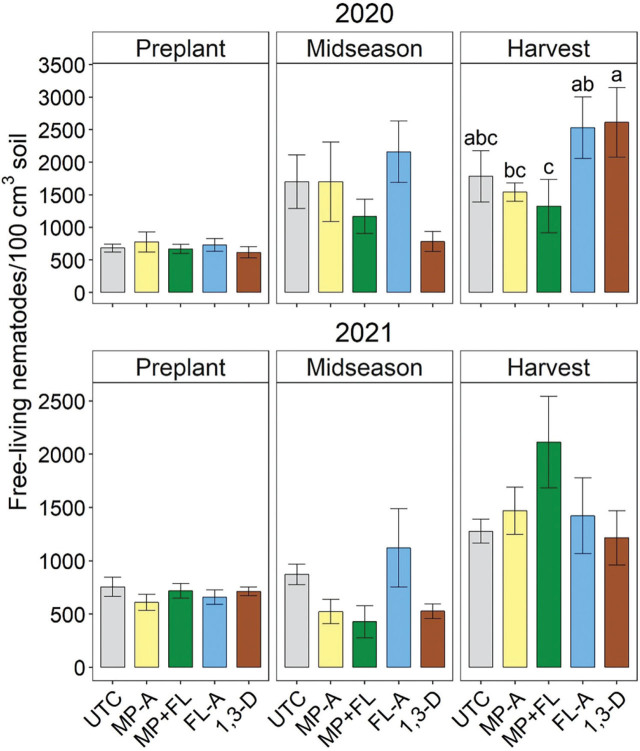
Free-living nematode soil population densities at preplant (before fumigation), midseason (37 to 44 days after planting), and harvest in 2020 and 2021 as affected by nematicide treatments. Bars and brackets represent means (N=6) and standard errors, respectively. “UTC”, “MP-A”, “MP+FL”, “FL-A”, and “1,3-D” represent untreated control, metam potassium alone, metam potassium mixed with fluensulfone, fluensulfone alone, and 1,3-dichloropropene nematicide treatments, respectively. Within each subfigure, means with different letters are significantly different (Fisher’s protected LSD, α=0.05).

Free-living nematode soil abundances were significantly affected by nematicides only at harvest in 2020 (Fig. 9). In that season, free-living nematode abundances were significantly greater following 1,3-D than MP-A or MP+FL and greater following FL-A than MP+FL.

## Discussion

The main objective of this study was to evaluate MP alone or in a mixture with FL for sting nematode management in potato production. Based on this study, any treatments with MP at the rate tested in this study (390 kg a.i./bedded ha) are not practical in the tested system due to risk of phytotoxicity. Metam potassium was phytotoxic to potato based on burn symptoms on shoots in 2020 as well as delayed emergence and reduced plant vigor relative to untreated in both years in this study. Phytotoxicity was generally less severe in 2021, with no qualitative symptoms (shoot browning) observed and plant stand and vigor impacts of a somewhat lesser magnitude. There was slight variation in severity of phytotoxicity between MP alone and in mixture based on some indicators, but symptoms were observed consistently enough in both treatments that it was clear that MP caused the phytotoxicity, rather than an interaction of FL and MP. Influence of MP treatments on potato yield was inconsistent, probably because MP both managed sting nematode abundances and was phytotoxic. Reduction of sting nematode abundances by both MP treatments suggests MP has potential for managing this potato pest, with no advantage of MP and FL mixture relative to MP alone, but phytotoxicity in this system must be resolved for either treatment to be practical.

Fumigants—including metam-based fumigants—are known to be phytotoxic to crops if the fumigant does not dissipate sufficiently before the crop is planted (Csinos et al., 2002; Desaeger et al., 2008). However, MP use is widespread in various crops—including some use in Florida potato production—and metam has not been phytotoxic in most previous studies (Ingham et al., 2007; Desaeger et al., 2017; Watson and Desaeger, 2019). So why was there phytotoxicity in this system and could adjustments be made to avoid phytotoxicity? Many factors can contribute to fumigant dissipation time and risk of phytotoxicity, including crop type (Csinos et al., 2002), fumigation rate (Ma et al., 2001; Klose et al., 2008), plantback time after fumigation (Csinos et al., 2002), application methods (Csinos et al., 2002; Desaeger et al., 2008), soil type (Ashley et al., 1963; Triky-Dotan et al., 2007), and environmental conditions at and following fumigation (Ashley et al., 1963; Gerstl et al., 1977; Desaeger et al., 2008). Based on these factors, phytotoxicity risk will vary by cropping system and these factors also suggest options for mitigation.

Regarding mitigation options, decreasing the MP application rate may decrease the risk of phytotoxicity and is the most practical option available in this cropping system. At a lower rate, there would be a lower initial fumigant concentration, so it should take less time to dissipate to a level that is not phytotoxic (Ashley et al., 1963; Klose et al., 2008). In this study, MP was applied at the maximum labelled rate (390 kg a.i./bedded ha), whereas, based on inquiries with local growers, around 170 kg a.i./bedded ha is more typical in potato production in northeast Florida (personal communication). However, to our knowledge, there are no peer-reviewed studies testing a reduced MP rate in this cropping system. Therefore, further research would be needed to determine if there is a MP rate—either alone or as a mixture with FL—in this system that is not phytotoxic but is still effective at managing sting nematodes.

Other factors, among those discussed above, may have contributed to phytotoxicity in this system, but it is generally either not practical or possible to control these factors. Additionally, for parameters that can be controlled (application methods, environmental conditions at fumigation, plantback time, etc.), instructions are listed on the fumigant product label and were followed, as is legally required for applicators. Increasing time between fumigation and planting could reduce phytotoxicity risk (Csinos et al., 2002), but anything longer than the 25-day period used in this study is typically not realistic. Similarly, in this study, tilling of beds during preplant fertilizer application as well as opening furrow for planting should have helped aerate the soil to dissipate fumigant residues, a common technique to mitigate risk of phytotoxicity. Conditions were abnormally dry after fumigation, particularly in 2020, and this system is largely dependent on rainfall to water in treatments, so dry conditions may have contributed to phytotoxicity. Typically, cool wet conditions increase time for metam salts and their derivatives to dissipate and decrease crop damage risk (Ashley et al., 1963; Gerstl et al., 1977). However, in this study, phytotoxicity was more severe in 2020—when conditions were very dry—than 2021. Phytotoxicity from metam salts under abnormally dry conditions has also been reported on cantaloupe (*Cucumis melo* var. *melo*) in central Florida (J.D. Desaeger, University of Florida, personal communication) and potato in Northeast Florida, via personal communication with local growers. This suggests extremely dry conditions are also a risk factor for phytotoxicity, although further research is needed to confirm and explain the mechanisms for this.

Fumigation with 1,3-D was the only consistently effective nematicide for managing sting nematode in this study considering both soil nematode abundances and potato yield. This is consistent with prior research and commercial practices as 1,3-D has typically been effective at managing sting nematode in previous studies (Weingartner and Shumaker, 1990; Crow et al., 2000a; Grabau et al., 2019) and it is a standard grower practice. Fumigation with 1,3-D was not as consistently effective at managing stunt nematodes, but this pest is not known to impact potato yield (Crow et al., 2000b).

Fluensulfone was not effective at managing sting nematode abundances in this study and provided an inconsistent yield return. In a previous study in Florida potato (Grabau et al., 2019), FL—at a similar rate to this study—was as consistently effective as 1,3-D for managing sting nematode abundances and increasing yield over a 3-year study. Environmental factors—particularly rainfall—may affect efficacy of FL and other nematicides, but rainfall and temperature varied by year and did not clearly explain differences in FL efficacy between those studies.

None of the nematicides tested had a consistent effect on free-living nematodes. Typically, fumigation with MP (Collins et al., 2006; Watson and Desaeger, 2019) or mixtures that include 1,3-D (Sanchez-Moreno et al., 2010; Timper et al., 2012; Watson and Desaeger, 2019) decreases free-living nematode soil abundances broadly across most trophic groups. In contrast, there is some prior information that FL has fewer non-target effects on free-living nematodes than other chemistries (Kearn et al., 2017; Waldo et al., 2019). Certain trophic groups, particularly fungivores, are often more sensitive to nematicides than other trophic groups (Fiscus and Neher, 2002; Watson and Desaeger, 2019; Grabau et al., 2020). Because only total free-living nematodes and not individual trophic group abundances were assessed in this study, it is possible that nematicides had undetected, negative impacts on individual trophic groups in this study. While infrequent, there are prior instances where fumigants had minimal impact on overall free-living nematode abundances (Desaeger et al., 2017). As a highly disturbed system with very frequent tillage and abundant inputs (fertilizers, pesticides, organic matter from crops), it is also possible that the nematode community in this system is composed predominantly of enrichment opportunists that rebound quickly after fumigation. More research would be needed to support that hypothesis.

In conclusion, MP at 390 kg a.i./ha, alone or as a mixture with FL, is not acceptable in this potato production system due to phytotoxicity from MP. Further research would be needed to determine if adjusting MP application parameters—particularly reduced application rates—could mitigate phytotoxicity while retaining effective sting nematode management in this cropping system. Based on this study, fumigation with 1,3-D continues to be the most consistently effective option for managing sting nematode in potato production. On its own, FL was not consistently effective for managing sting nematode despite consistent efficacy in prior research. Nematicides did not have a negative impact on the overall free-living nematode community, but further research would be needed to assess impacts on individual groups of free-living nematodes in this system.
